# AGENTi: APCI-ESI Analyte Ionization Fidelity Pre-Screening Workflow using Multimodel Inference

**DOI:** 10.1021/acs.jcim.6c01085

**Published:** 2026-09-24

**Authors:** Mithony Keng, Kenneth M Merz

**Affiliations:** † Department of Chemistry, 3078 Michigan State University, East Lansing 48824, Michigan, United States

## Abstract

Ionization sources convert neutral analytes into ions, enabling their subsequent separation and detection by mass spectrometry. Depending on the technique, this mechanism occurs within a solution or in the gas phase. The two most common methods to generate ions are electrospray ionization (ESI) and atmospheric-pressure chemical ionization (APCI), respectively. Both ESI and APCI can generate the same resultant ion species; however, it has been shown that preferential charge sites can change in response to an ionization source environment. Therefore, an additional quality control is warranted *a priori* to delineate erroneous analyte identification and/or unequivocal structural assignment. For protonation, protomer fidelity can be assessed by comparing the ion-mobility collision cross section (CCS) values obtained for both ESI and APCI. In this work, we utilized proxy standards (i.e., controls) derived from the available literature to develop a proof-of-concept multimodel inference application called AGENTi to probe differences in ionization for high-throughput protomer shift exploratory analysis. AGENTi uses in sequence the TensorFlow Autoencoder anomaly detection and the YDF Gradient Boosted Tree machine learning frameworks to generate more balanced and robust anomaly scores. We found that using a multimodel screening approach can reduce false positive capture at an acceptable loss of true positives. Moreover, we expect to achieve a better recovery of true positives with an improvement on the current Gradient Boosted Tree regression model for ΔCCS prediction as more ESI-APCI dual data are obtained for training. In all, we demonstrated in this pilot study that our foundation model for *a priori* probing of ionization pathway fidelity is feasible and efficient with AGENTi. The AGENTi prototype application and documentation are accessible at https://github.com/mitkeng/AGENTi.

## Graphical abstract

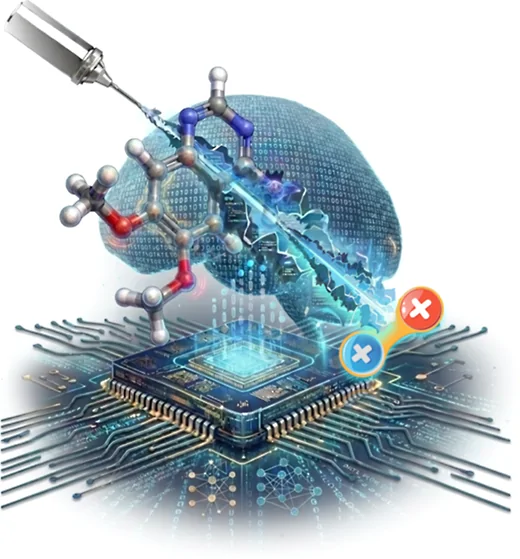

## Introduction

Drift-tube ion mobility mass spectrometry (DTIM-MS) is a technique within the mass spectrometry family of technologies that can be used to separate and detect chemical analytes as a function of mass-to-charge (*m*/*z*) and drift mobility under a uniformly weak electric field. DTIM-MS is especially practical for separating analytes with identical *m*/*z* (i.e., isomers) that have distinct conformational sizes, which, in effect, can result in differential ion-neutral collision mobilities. Most analyte samples, however, are initially charge-neutral prior to a DTIM-MS experiment and therefore require preprocessing for conversion into ions before they are introduced into field-induced drift tubes for signal detection.^1^

For ion generation, two of the most common ionization methods utilized in mass spectrometry are electrospray ionization (ESI) and atmospheric-pressure chemical ionization (APCI). The main distinctions between these two methods are that the source of ionization generally occurs in solution for ESI and in the gas phase for APCI.^2^ Importantly, the consistency of ionization products across ESI and APCI has not been thoroughly studied. This is a valid concern because preferential charge sites can change in response to different ionization environments, which consequently can lead to inconsistent experimental results.^3–6^ Additionally, *a priori* knowledge of analyte charge state behavior can help to delineate incorrect analyte identification and/or unequivocal structural assignment. This discrimination is not only crucial for experimental reproducibility but also for establishing predictive computational workflows that rely on chemically specific experimental reference.

For DTIM-MS experiments that involve protonated adducts (e.g., [M + H]), protomer fidelity can be audited by comparing the ion-mobility collision cross section (CCS) values obtained for ESI and APCI.^7–12^ Based on our survey of more than 600 reference analytes with both ESI- and APCI-derived CCS values reported by Baker and co-workers,^13,14^ which show an average difference of ∼0.3% (±0.8% standard deviation), we conservatively propose that identical protomers must exhibit a CCS difference (ΔCCS) of less than 2% between the two methods. We determined this by considering that 95% of data points for a normal distribution are within two standard deviations (σ) of the mean, which, in accordance, would make any data point with a ΔCCS of <1% nominal. We adjusted this initial nominal threshold for our training data selection to 2% since isomers have been observed to exhibit ΔCCS of roughly ≥2%, with constitutional isomers (protomers) having a range of ∼1–3% (with a mean of ∼2%).^15,16^ Moreover, Miller et al. obtained an average ΔCCS of ∼3% between N-protomer and O-protomer for seven fentanyl analogs, with each analog protomer pair having a ΔCCS >2%.^17^ Similar work by Ieritano et al. obtained an average ∼1% ΔCCS for their fentanyl analogs, with β-hydroxy thiofentanyl having 2% ΔCCS between N- and O-protomer.^18^

We therefore determined that it was highly appropriate that data points or systems that generated ΔCCS ≥2% are not nominal for this work, and we can justifiably flag them as potential ionization anomalies. Furthermore, interlaboratory experimental CCS reproducibility and repeatability were found to be within 2% for identical ions.^19–24^ Accordingly, we found within the same reference dataset five systems (see Table 1) that have ΔCCS above the 2% threshold, which we will use to validate our proof-of-concept method for protomer divergence probing using a multimodel artificial intelligence approach. Since these outlier systems have not been experimentally verified to exhibit ionization species divergence, they are designated only as proxy controls in this article moving forward.

**Table 1:** Five Proxy Anomaly Controls Used to Fine-Tune the Selectivity-Specificity Ratio and Validate the Multimodel Performance at Capturing Anomalous Chemicals

[M + H] anomaly controls	APCI CCS (Å^2^)	ESI CCS (Å^2^)	%Δ CCS
4,4′-methylene bis	179.1	171.2	4.5
denatonium	180.2	188.6	4.6
diflufenican	184.7	189.1	2.4
*m*-CRESIDINE	124.8	135.7	8.4
tyrphostins	177.3	171.0	3.6

It should be noted that building an effective machine-learning model for anomaly detection on limited data is feasible since a typical anomaly algorithm focuses on identifying uncommon patterns or outlier relationships. Thus, in this work, we utilize proxy anomaly standards (i.e., controls) to develop a multimodel inference application called AGENTi to screen for the incidence of ionization divergence between the ESI and APCI ionization methods. To achieve this, we will implement machine-learning frameworks involving neural networks and regression to screen for anomalies and predict the change in CCS between ESI and APCI, respectively.

It is important to emphasize that this integrated pipeline functions as an upfront, high-throughput computational screening framework designed to maximize the utility of potentially large-scale datasets rather than serving as an empirical characterization study. In predictive discovery science, leveraging established repositories is foundational for training machine-learning architectures capable of mapping subtle molecular trends. AGENTi addresses this paradigm by acting as an upfront, digital quality control filter before the commitment of laboratory resources. Ultimately, this work presents a transferable cheminformatics methodology focused on proactive anomaly detection, establishing an automated pipeline for cross-source data auditing and enhancing overall data quality control.

## Computational Method

### Feature Engineering

Figure 1 shows the overall programmatic workflow executed to achieve an optimal AGENTi version for effective APCI-ESI ionization anomaly probing. The core innovation of the AGENTi data structure and feature engineering is the 18-dimensional mixed manifold framework. It integrates 12 2D structural features with six 3D conformational and electronic features to capture comprehensive molecular fingerprints. The 2D descriptors consist of TPSA to track the topological polar surface area, VSA to quantify molecular bulk via van der Waals surface area, and double and triple bond counts for electronic signature. It also includes the features heteroatom count, halogen count, ring count, positive and negative charge count, and LogP to measure lipophilicity. The 2D set is completed with positive and negative charge counts, along with the ad hoc parameter features, radii, and mass ( S ) and electronic properties ( E ), which capture custom steric and electronic distribution qualities. Moreover, eqs 1 and 2 are used to compute S and E .

1 $$S=\frac{n\sum_{i=1}^{n}r_{i}}{\sum_{i=1}^{n}m_{i}}$$

2 $$E=\frac{\sum_{i=1}^{n}{Ι}_{i}\sum_{i=1}^{n}\chi_{i}}{n\sum_{i=1}^{n}\alpha_{i}}$$

for the above equations, *i* is the ith atom, *r* is the atomic radius, *m* is the atomic mass, Ι is the ionization energy, χ is the electronegativity, α is the polarizability, and *n* is the total number of atoms in a system. These features were used to train AGENTi to generate an overall anomaly score.

**Figure 1:**
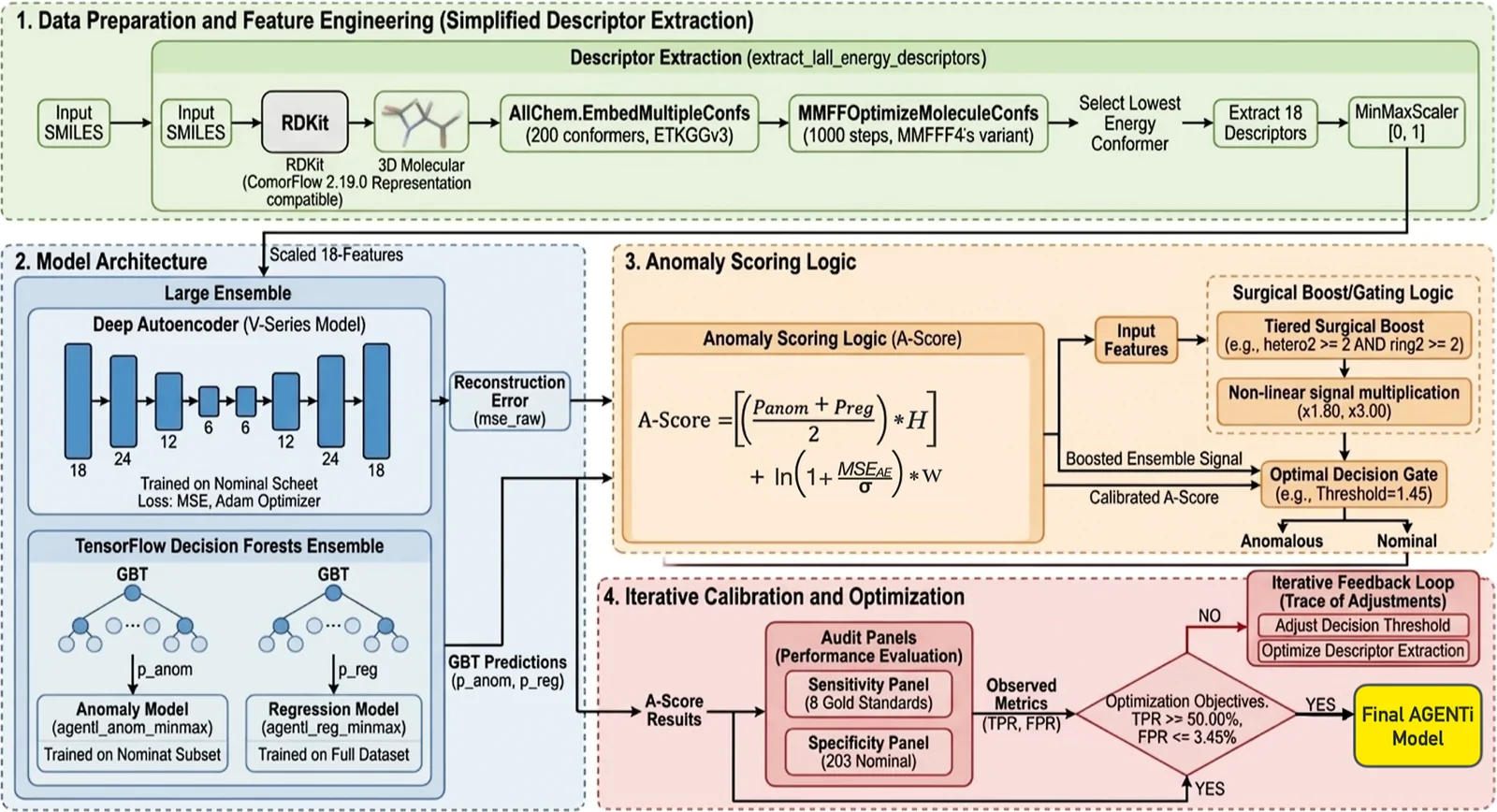
Operational schematic of the AGENTi ionization anomaly screening model. Step 1 initiates 18 mixed descriptor extraction from 2D and 3D sources. In step 2, normalized features are evaluated by three parallel models to generate distinct intermediate outputs for downstream processing. Step 3 integrates these intermediate outputs into a mathematically blended anomaly scoring metric (A-Score) modulated by weighting and scaling factors. Model optimization concludes in step 4, where final classification results are evaluated against target performance constraints to guide the post-hoc calibration of hyperparameters.

The six 3D descriptors used to expand our training manifold include dihedral to track torsional angle distribution, dipole moment, and hetero_com_dist to evaluate the geometric distance from heteroatoms to the molecular center of mass, radius of gyration, solvation energy, and tSNE to provide a low-dimensional spatial representation of conformational variance.

For 3D-based descriptor extraction, RDKit (compiled with TensorFlow 2.19.0) executed a deterministic conformer search. We initially generated 200 conformers using the ETKDGv3 algorithm with fixed random seeds to eliminate stochastic variance (see Figure 2). The resulting coordinates underwent force-field refinement with 1000 steps using MMFF94. The pipeline then filtered the complete ensemble to isolate the global lowest-energy conformation, which effectively minimized stochastic drift and ensured reproducible descriptor calculations. Physics-based numerical calculations were integrated to derive the solvation energy and dipole moment directly from the lowest-energy state. It is worth noting that the 3D generation step does require a higher computational cost than calculating standard 1D and 2D topological fingerprints directly from SMILES strings. However, this method remains optimized for high-throughput molecular library prescreening because the protocol relies on rapid, classical force-field methods rather than expensive, high-level QM calculations like density functional theory.

**Figure 2:**
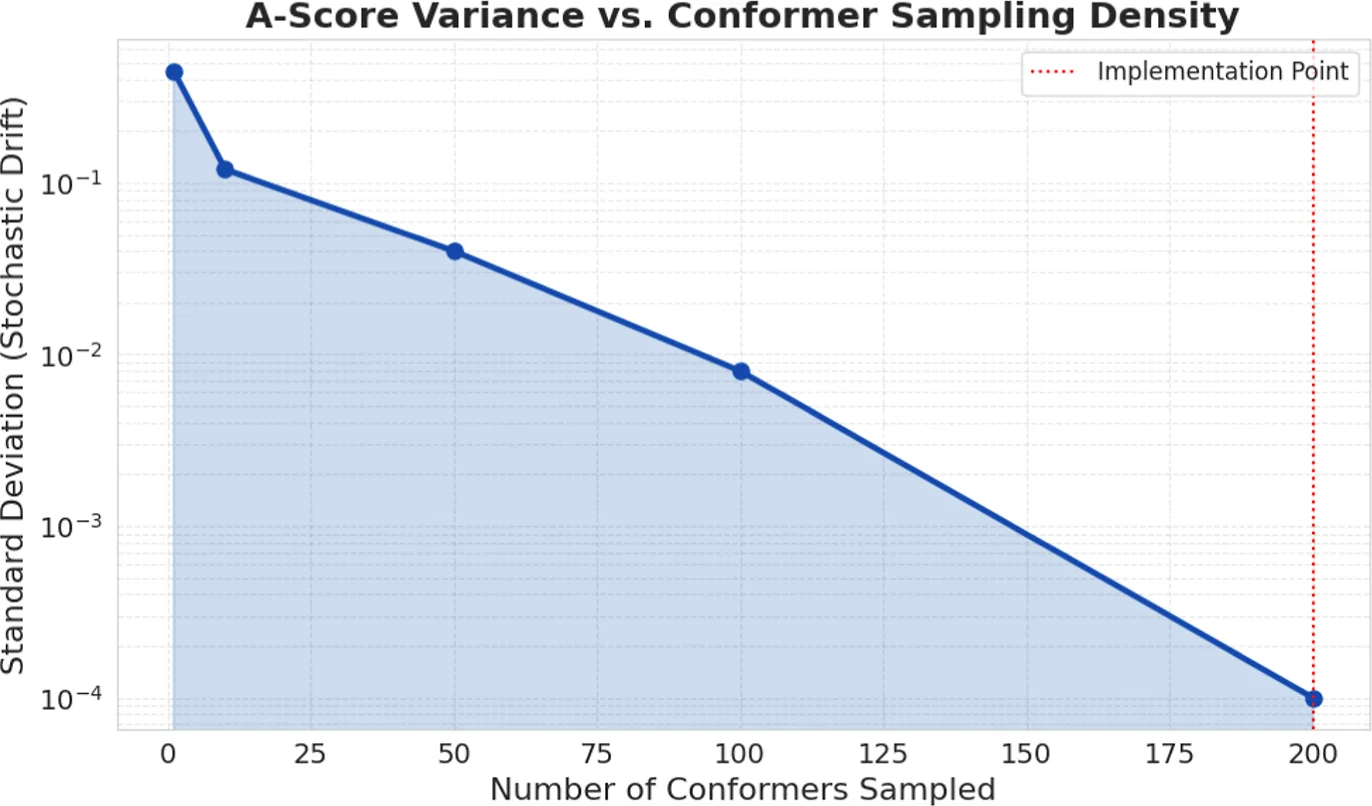
Minimizing A-score variance through conformer ensemble size sampling. Low-density sampling (*n* < 10) results in high A-Score instability (Std Dev >0.40) due to different energy minimum conformers for 3D descriptor calculations. Implementation of 200 conformers per molecule achieves result stability with a standard deviation of ∼0.0001.

A standard MinMaxScaler was applied to scale all 18 extracted features into a strict numerical bound of [0,1]. The scaler is fitted exclusively on the training dataset to establish normalization boundaries. Locking these boundaries post-fit ensures that subsequent inferences are fully insulated against test set data leakage across both the neural network and decision forest architectures.

### AGENTi Architectures

AGENTi implements a hybrid ensemble architecture that combines an unsupervised deep neural autoencoder (TensorFlow Keras^25^ autoencoder algorithm) with the YDF ML frameworks for anomaly detection. First, the deep autoencoder compresses and reconstructs the 18-dimensional feature space, establishing a baseline for anomaly detection on the reconstruction error. The network starts with an 18-dimensional input layer that feeds into encoder dense layers of 24, 12, and 6 neurons by using the ReLU activation function. The network uses mean squared error (MSE) as its loss function and the Adam optimizer with 100 epochs at a fixed batch size of 32. Training was restricted to a nominal subset of the training data.

The YDF models consist of the gradient-boosted tree (GBT) configured for supervised regression tasks and the isolation forest (IF) for unsupervised anomaly scoring. The hyperparameters for the GBT model are shown in Figure S1 in the Supporting Information. These two models are used to evaluate the 18-descriptor normalized manifold in parallel with that of the deep autoencoder.

### Training Regimen and Logic

A dataset consisting of 603 chemical systems with dual APCI and ESI CCS values was used for training the models (for a complete list see Table S1 in Supporting Information). We scrutinized the protomer/ionization fidelity of a chemical and, therefore, its intrinsic nominal data points, by calculating the ΔCCS between the APCI and ESI experimental CCS values. More precisely, the three anomaly scoring models were trained on data points of 603 chemicals (see Figure 3) that have ΔCCS <2%. Again, we initially calculated *a* ∼1% nominal threshold by adding two times the standard deviation to the average ΔCCS (i.e., ΔCCS + 2σ) for the total reference data^13^ but increased the threshold to 2% to accommodate precedented experimental findings.

**Figure 3:**
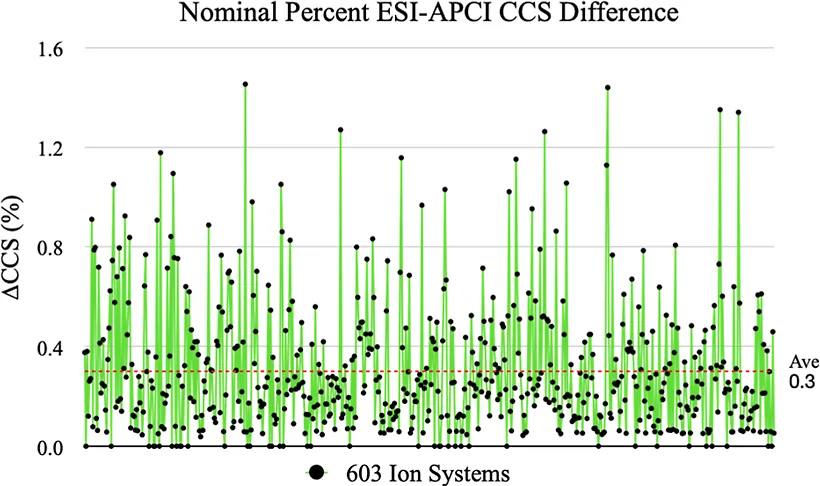
ΔCCS values (mean ≈ 0.3) for the 603 nominal chemical systems used to train the autoencoder anomaly model to establish a baseline for normal data points. The 0.3% mean relative percent difference sets our nominal baseline.

APCI-ESI source ΔCCS discrepancies do not arise solely from altered protonation sites and can be attributed directly to the difference in internal energy distributions generated by the higher temperature used in APCI for desolvation, leading to the activation of high-energy barrier conformational transitions that are not favorable under ESI conditions. This effect can be independent of the charge localization site, introducing localized discrepancies in ΔCCS without charge migration. For instance, Valadbeigi and Causon (2024) showed in their work that monoprotonated chloroquine ([M + H]+) is populated in the gas phase as an equilibrium mixture of an extended conformer (conformer a2, Δ*G*_gas_ = 0.0 kJ mol^–1^, 65.6% populated) and a compact conformer (conformer a7, Δ*G*_gas_ = 1.6 kJ mol^–1^, 34.4% populated).^26^ Despite sharing an identical protonation site (protomer a), these distinct thermal energy profiles yield a ΔCCS of 5.9%.

Accordingly, the AGENTi autoencoder architecture was trained using a conservative 2% nominal descriptor boundary to mitigate false-positive anomalies arising from such—but not limited to—thermally driven structural fluctuations within identical ion species. This boundary establishes a buffer region within the learned latent space against minor conformational variations to effectively clear nominal chemical spaces where the global background mean relative percent difference discrepancy is small at ∼0.3% (±0.8% standard deviation, σ). Consequently, anomaly flags are reserved strictly for extreme data point outliers.

### Anomaly Scoring

The autoencoder algorithm uses the 18 features extracted from the input SMILES and scales the data points using the MinMaxScaler to yield values in the range of 0 and 1. Subsequently, the decoder generates a prediction of the original data points, which leads to a reconstruction error, MSE_AE_, between the input and output. From the list of reconstruction errors generated from the nominal training data (i.e., *N* = 603), a standard deviation, σ, is obtained and used as a constant in our anomaly equation for evaluating test systems moving forward. Both MSE_AE_ and σ can be calculated using the following equations

3 $${MSE}_{AE}={\left(x_{i}-{\mathrm{x̂}}_{i}\right)}^{2}$$

4 $$\mu =\frac{1}{N}\sum_{i=1}^{n}{MSE}_{AE}$$

5 $$\sigma =\sqrt{\frac{1}{N-1}\sum_{i=1}^{N}{\left({MSE}_{AE}-\mu \right)}^{2}}$$

for eqs 3–5, *x*_*i*_ is the encoder input value for feature *i*; ${\mathrm{x̂}}_{i}$ is a value (hypothesis) generated by the decoder for data point *i*; *N* (*N* = 603) is the total number of data points or training examples; μ is the mean of the dataset of reconstruction errors. For our current training set, σ is 0.058.

Similarly, the same quantity (i.e., *N* = 603) and quality (i.e., 18 features) of training data were also used to train the YDF IF anomaly scoring model and the GBT regression model. The hyperparameters for IF were maintained at default settings. Altogether, the overall anomaly value, *A*(*x*), per data point as a function of an input vector of 18 features, can be calculated using eq 6

6 $$A\left(x\right)=\left[\left(\frac{P^{\text{IF}}\left(x\right)+P^{GBT}\left(x\right)}{2}\right)\times H\left(r\right)\right]+w\times \ln\left(1+\frac{{MSE}_{AE}\left(x\right)}{\sigma }\right)$$

the term on the left is the core ensemble. It is computed as the average of the isolation forest model, *P* ^IF^, and the GBT regression model, *P* ^GBT^. Within AGENTi, a stepwise structural multiplier, *H*(*r*), scales the anomaly signal based on the molecular ring count (*r*), This multiplier accounts for complex topologies using the following conditional logic

$$H\left(r\right)=\{\begin{array}{l} 3.0\;if\;r\geq 3\\ 1.8\;if\;r=2\\ 1.0\;otherwise \end{array}$$

we used the ring count (*r*) for the structural multiplier, *H*(*r*), to align the scoring function with volatile chemical profile and model performance. Analytically, the baseline ensemble models tend to under-score compounds with rings by misclassifying them as nominals even when their novelty is high. The multiplier acts as a precise counter-gain to fix the conservative bias. This adjustment effectively forces unusual, ring-heavy positive controls such as denatonium, diflufenican, and quinine to correctly cross the anomaly threshold. The additive term on the right, ${MSE}_{AE}\left(x\right)={∥x-\mathrm{x̂}∥}_{2}^{2}$ , represents the mean squared error of the feature reconstruction (i.e., reconstruction error), where x̂ is the reconstructed feature vector. A natural logarithm regularizes the reconstruction error, which was calibrated by using the standard deviation σ = 0.058 and weighted by *a* factor *w* = 0.35.

For anomaly screening, the resulting *A*(*x*) is compared against a global anomaly threshold. We initially fine-tuned our model for peak anomaly threshold efficiency by running a manual grid search across the parameter range of 0.8 to 1.4 (incremented in steps of 0.2). To evaluate each threshold candidate, we implemented a validation strategy benchmarked against our proxy anomaly control dataset. Consequently, we found an initial threshold value of 1.40 to be a good starting candidate for the AGENTi operational qualification testing.

### Post-Hoc Calibration and Threshold Optimization

A two-phase hyperparameter optimization routine was implemented to systematically establish a robust decision boundary and insulate the classification logic from human bias. The global classification gate was calibrated by benchmarking model outcomes against an independent validation dataset (*N* = 211) containing five proxy-positive anomaly controls and 206 nominal negative background compounds (negative controls). An initial coarse grid search was executed across the parameter domain of 0.80 to 1.40 in steps of Δ = 0.20 to isolate the operational baseline of the anomaly value *A*(*x*). This sampling approach is to identify where extreme structural outliers naturally segregate from the nominal population. A threshold of 1.40 emerged as the peak preliminary candidate, but manual selection lacked objective scaling constraints and necessitated a post-hoc numerical calibration. The hyperparameter tuning was framed as a constrained optimization problem to finalize the model configuration. The primary objective was regulating the contribution weight (*w*) of the log-regularized autoencoder reconstruction loss relative to the decision forest architectures, while simultaneously setting a definitive global decision threshold. The objective function was bounded by the following performance constraints

targetsensitivity(TPR)≥50%

targetfalsepositiverate(FPR)<5%

The tuning algorithm iteratively sampled the lower-bound threshold domain from 1.25 to 1.50. The optimal configuration converged at an autoencoder reconstruction weight factor of *w* = 0.35 and a global decision gate of τ = 1.45, satisfying the objective function

$$classification=\{\begin{array}{l} anomalous,,if\;A\left(x\right)\geq 1.45\\ nominal,if\;A\left(x\right)<1.45 \end{array}$$

Evaluating the validation dataset under the optimized τ = 1.45 gate demonstrated that the model effectively minimized false positive flags without a critical loss of true positive capture. The threshold calibration works in tandem with an automatic multiplication step to prevent the model from missing unusual chemical spaces. Under this scoring rule, when an input compound has two or more heteroatoms and two or more ring structures, its score is multiplied by either 1.8 or 3. After this multiplication step, the final scores are sent directly to the calibrated classification gate. We benchmarked the model’s classification performance against a randomized chemical space to evaluate the statistical significance of AGENTi’s anomaly detection under limited data constraints. For this task, we subjected our model to 10,000 Monte Carlo simulations utilizing the Python SciPy libraries to establish a random chance baseline. A hypergeometric distribution test evaluated the statistical significance by modeling the probability of the observed Monte Carlo anomaly discovery rate. This process calculates an exact *p*-value, establishing whether AGENTi’s predictive capabilities are derived from feature learning or are an effect of stochastic artifacts and background data noise.

It was also necessary to evaluate the optimized 1.45 global threshold for confirmation bias in the chemical spaces of the controls. We therefore challenged our model’s generalization via structural similarity testing using an independent evaluation set consisting of 1000 two-heteroatom systems that were randomly generated using RDKit. Pairwise similarity was quantified using the 2048 bit Morgan fingerprints (radius = 2) to evaluate structural overlap against our proxy controls before anomaly performance analysis. A pairwise Tanimoto similarity score of >0.50 indicates a structurally similar compound pair.

## Results and Discussion

Prior to evaluating the performance of AGENTi for APCI/ESI ionization anomaly screening, the model’s internal decision-making logic was assessed using a global KernelSHAP framework mapped relative to AGENTi’s Autoencoder’s reconstruction loss to ensure interpretability. The initial proxy anomaly controls were utilized to establish the SHAP background baseline. Here, we use SHAP (SHapley Additive exPlanations) values to quantify the contribution of each structural descriptor to the individual instance’s deviation from the expected reconstruction error baseline. This allows the architecture to classify inputs as anomalous or nominal based on the magnitude of this drift. As shown in the SHAP summary plot for positive controls (Figure 4), the features are organized vertically by their overall global importance, illustrating how the model constructs its decision boundaries around interpretable physicochemical properties rather than relying on uninterpretable background noise.

**Figure 4:**
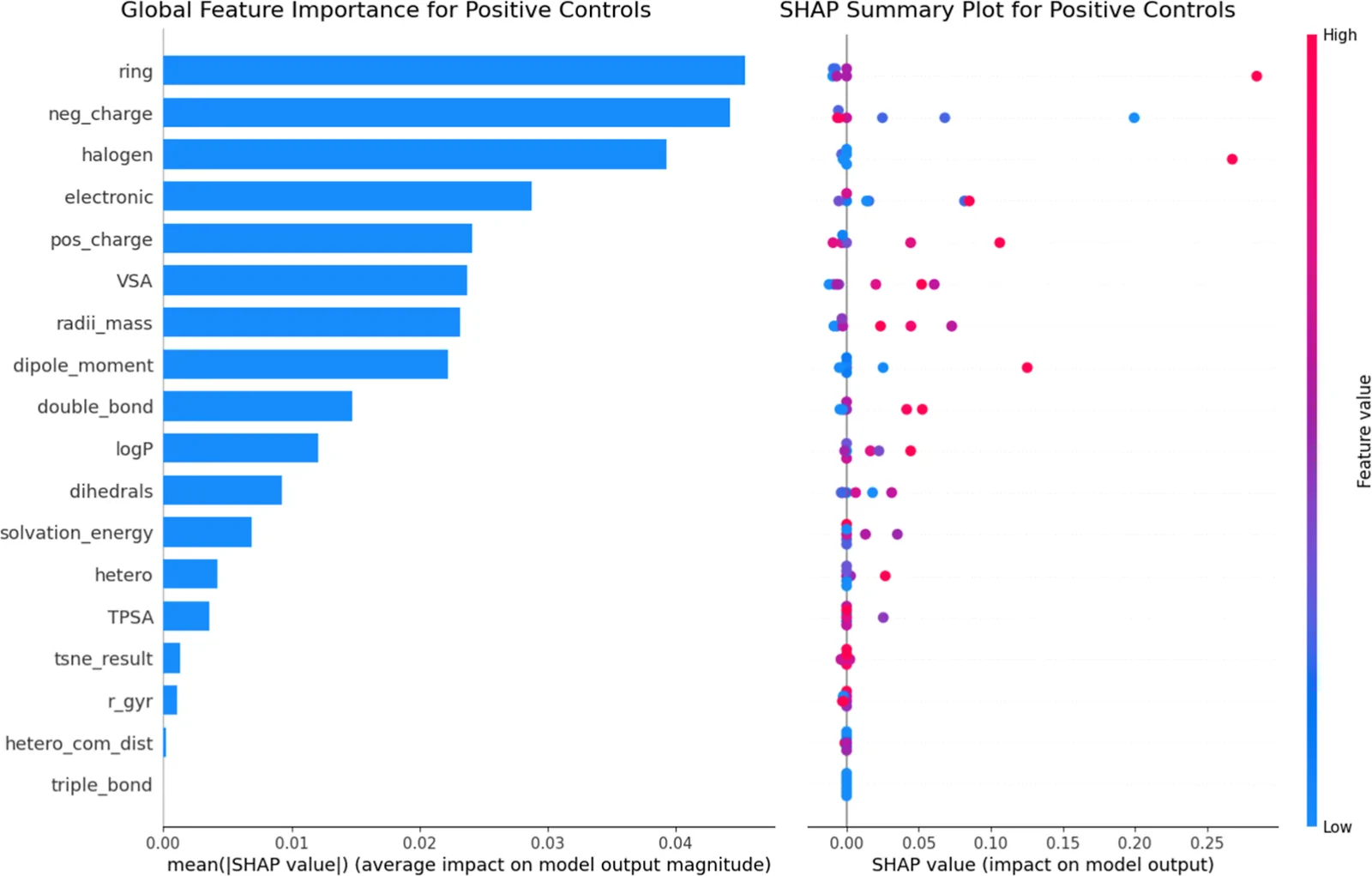
(Left) Global feature importance profile for positive controls. Features are ranked vertically based on their mean absolute SHAP value, representing the average impact on model output magnitude. Structurally dominant descriptors including ring count (ring), negative charge (neg_charge), and halogen count (halogen) emerge as the primary global drivers of the model’s decision-making logic, while hetero_com_dist and triple bond count (triple_bond) exert minimal global influence. (Right) SHAP beeswarm plot illustrating feature value distributions across positive controls. Features are ordered vertically by their global predictive importance from top to bottom. Individual data points represent a single sample, with horizontal positioning indicating the SHAP value (impact on model output) and color representing the relative feature value (magenta/red for high values, blue for low values). Accumulations of high feature values in the positive *x*-axis (>0.00) denote descriptors that consistently drive elevated reconstruction loss, establishing a relationship between structural variations and anomaly detection thresholds.

**Figure 5:**
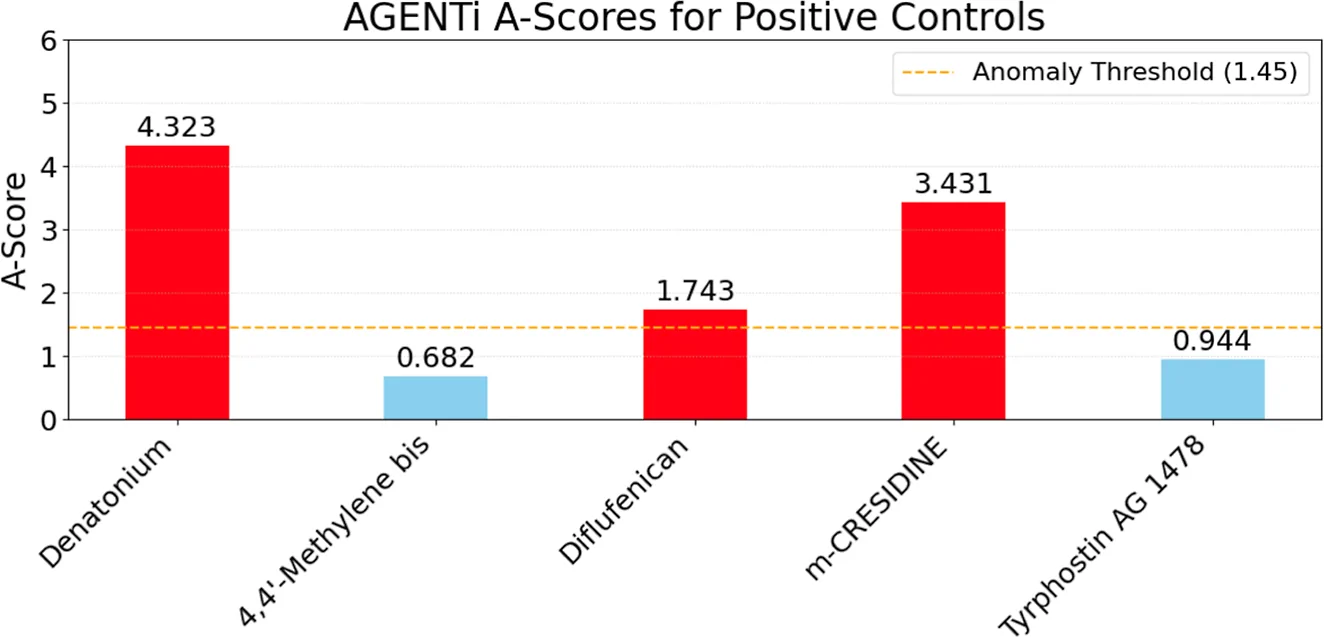
AGENTi predicted A-score values for proxy-positive (anomaly) controls. Red bar indicates anomalous (ionization diverging) under the current global anomaly threshold. The operational anomaly threshold value is 1.45 (orange dashed line).

The SHAP results also reveal a clear mechanistic alignment between high feature values and elevated anomaly scores. For the highest-ranked descriptors at the top of the profile, a high feature value directly correlates with large, positive SHAP values that extend beyond 0.25. This distribution demonstrates that elevated expressions of these primary topological and electronic descriptors serve as the dominant drivers to push the autoencoder’s reconstruction loss away from the nominal baseline. Conversely, low feature values cluster tightly around a SHAP value of 0.00, indicating that baseline levels of these descriptors exert negligible impact on anomaly generation. This clear directional separation provides strong evidence that AGENTi operates as a chemically intuitive filter, successfully flagging structural anomalies tied to feature attribution prior to final threshold evaluation.

For ionization species divergence testing, the generated anomaly value, *A*(*x*), is evaluated against our 1.45 global threshold. We obtained this *a priori* from the validation set containing chemicals with both nominal and anomalous ΔCCS through AGENTi. We arrived at the 1.45 value after iterative rounds of threshold and/or post-hoc hyperparameter recalibration that resulted in optimal detection of our proxy controls that reciprocated a favorable FPR/TPR ratio. We found that systematically increasing the threshold using grid-search optimization from 0.8 to 1.40 decreases the detection sensitivity, which corresponds to a loss of true positives or, alternatively, a gain of false negatives for the anomaly controls. Consequently, applying a 1.45 decision threshold to the validation dataset (*N* = 211) enabled AGENTi to isolate three out of five positive controls—specifically, denatonium, diflufenican, and *m*-cresidine as ionization anomalies (see Figure 5). This evaluation yielded an overall classification specificity of 97.54% (FPR = 2.46%) and a sensitivity of 60% (TPR = 60%).

Upon investigating the structural failure modes, we reason that the specific physicochemical nuances behind the remaining 40% false negative rate are likely driven by the model’s reliance on neutral molecular profiles. Tyrphostin AG1478 serves as a prime example of this limitation, generating a sub-threshold A-score of 0.944. This low score occurs despite the compound possessing a significant empirical cross-source ΔCCS of 3.68% (APCI = 177.3 Å^2^ versus ESI = 171.0 Å^2^). AGENTi extracts its 18 structural and conformational descriptors exclusively from the native, unprotonated form of the input SMILES. Tyrphostin’s native structure possesses typical spatial properties, mapping seamlessly within the feature space distribution of the 603 background chemicals. The autoencoder reconstructs these baseline neutral properties with low error. This minimal baseline reconstruction loss keeps the final score sub-threshold, even though the compound satisfies the topological criteria for the *H*(*r*) multiplier.

Alternatively, *m*-cresidine, denatonium, and diflufenican have physical or electronic properties within their native states that contrast sharply with the nominal background population. Denatonium features a quaternary ammonium with a localized positive charge center. This feature generates a large reconstruction discrepancy within the autoencoder’s latent layer. Diflufenican introduces is halogenated that skews the standard relationship between molecular weight and radius of gyration. This yields a high reconstruction error that is further amplified by the 3.00 structural multiplier. For *m*-cresidine, the small molecular size and compact aromatic layout position it at the extreme lower boundary of the training manifold, preventing accurate reconstruction. These distinct variations provide sufficient numerical signal to trigger the anomalous classifications. Nevertheless, the real-world experimental ionization divergence is driven by post-ionization charge migration liabilities that remain completely invisible to a purely neutral descriptor manifold.

Next, we profiled a randomly generated set of 1000 two-heteroatom molecules against a Tanimoto similarity (Morgan fingerprints) threshold of 0.5 to evaluate whether high structural similarity to positive controls drives model outcomes or introduces confirmation bias (Figure 6). The results reveal that most of the background population clusters densely between 0.0 and 0.2, establishing a clear structural separation from the controls. Of the 1000 compounds, only six exceeded the 0.5 Tanimoto similarity threshold. Moreover, beyond this threshold, only one out of six was flagged as anomalous, yielding a local positive hit rate of 16.67% compared to an overall baseline FPR of 2.46%.

**Figure 6:**
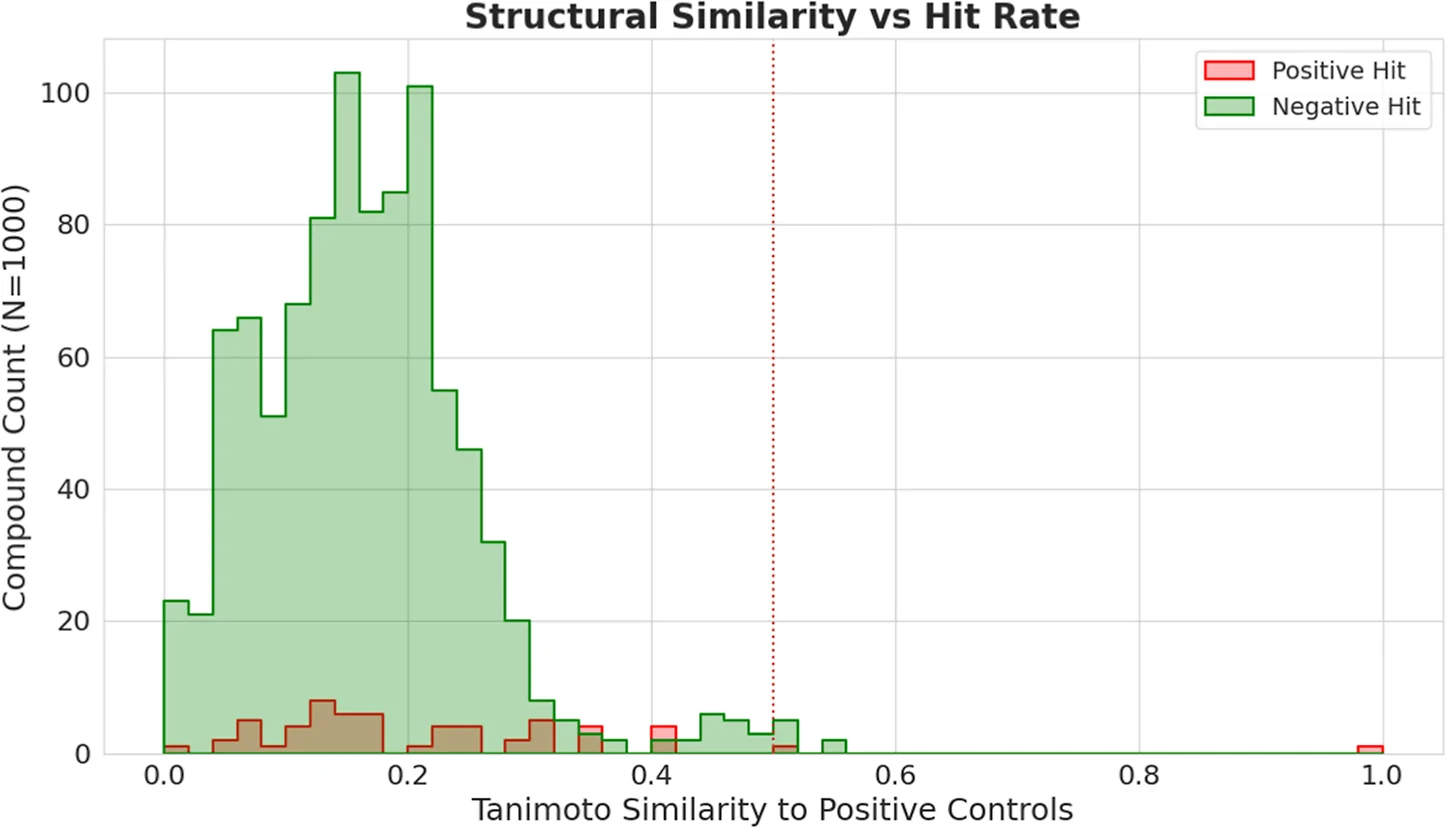
Impact of structural similarity on the model positive hit rate. Histogram shows background compounds (*N* = 1000) against Tanimoto similarity to positive controls using Morgan fingerprints. Green denotes negative hits (nominal compounds). Red denotes positive hit classifications. The vertical dotted line marks the Tanimoto threshold (0.5). Six out of 1000 compounds have >0.5 similarity with controls. One compound with >0.5 similarity has a positive classification (16.67% hit rate).

This comparison evaluates a common issue of whether the model overfits to the chemical space of the proxy controls. While the positive hit rate scales due to the small sample size within the highly similar structures, the absolute metric reveals that five out of six highly similar compounds were successfully cleared. If the model suffered from systematic confirmation bias, the anomaly hit rate would scale proportionally with proximity to the positive controls, leading to a large failure across the >0.5 Tanimoto zone. However, since most of these outliers were cleared, this demonstrates that structural proximity to positive controls does not act as an automatic driver of anomalous outcome. This clear latent separation allows us to map the uncertainty and decision boundary of the hybrid ensemble. The variance in individual intermediate models, specifically the deep autoencoder’s reconstruction error (σ = 0.058) versus the GBT predictions, is effectively regularized by the log transformation in the scoring logic. This strategy prevents categorical overfitting and stabilizes predictions across varied chemical spaces.

The similarity results complement the physicochemical descriptor distributions observed in Figure 7A–D. While the Tanimoto similarity demonstrates that the background compounds are structurally distinct from the controls, Figure 7 highlights that the true drivers of AGENTi’s decision are localized, nested physical boundaries. Specifically, anomalies are situated in narrow, nominal sub-spaces such as 100–200 molecular weight (Figure 7A) and the 1.0–2.0 logP range (Figure 7B). By verifying that our classification logic does not rely on superficial topological proximity, we confirm that our calibrated 1.45 decision threshold captures authentic anomalous signatures based on intrinsic, physical properties rather than 2D structural trends.

**Figure 7:**
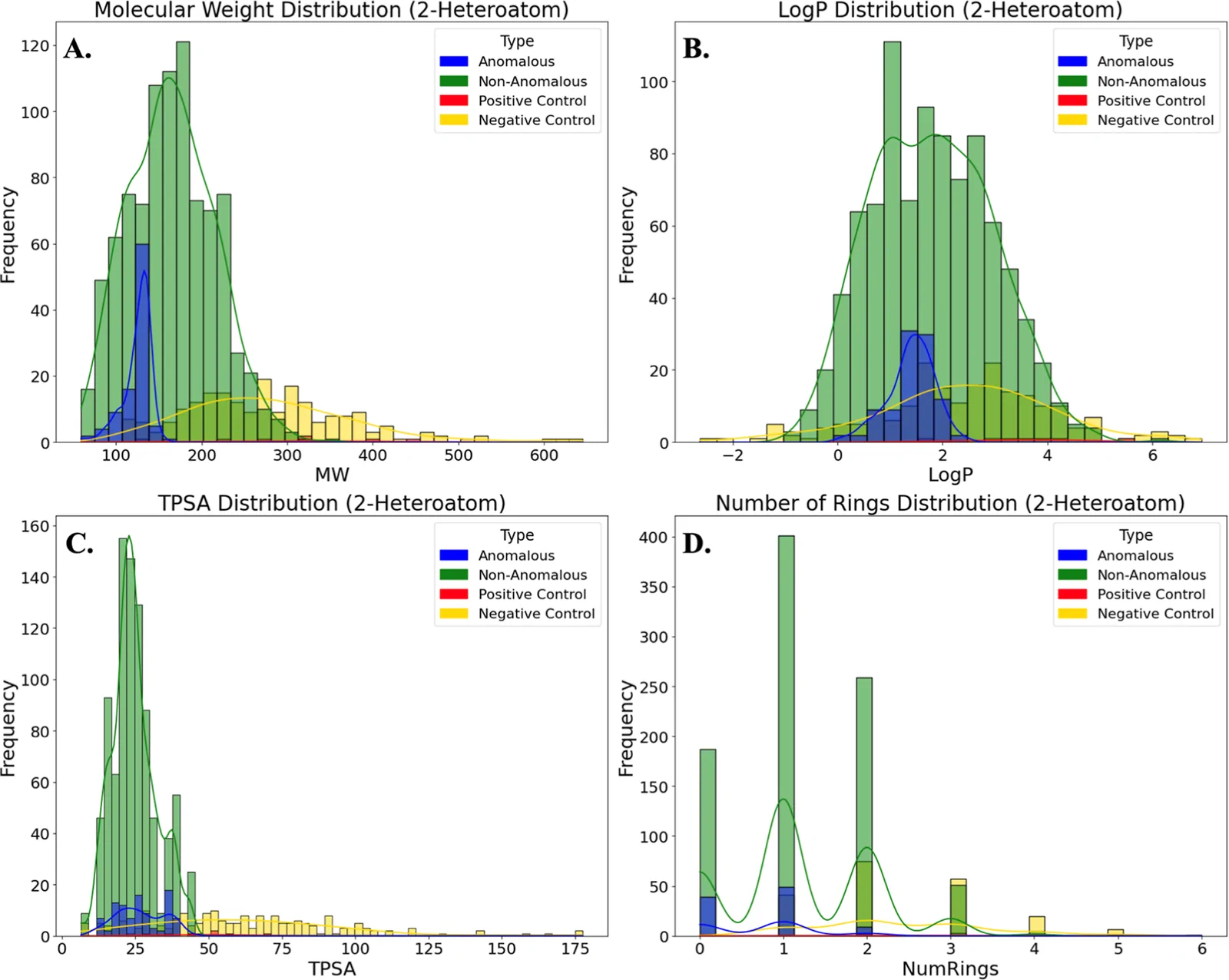
Population distributions of descriptors across nominal and anomalous structural spaces for the 2-heteroatom compounds test set. (A) Molecular weight distribution showing a localized cluster of anomalous instances (blue) nested within the lower-bound range (∼100–130 MW) of broader non-anomalous population (green). (B) logP distribution demonstrating that anomalous structures occupy a restricted, nominal lipophilicity window (1.0–2.5 logP). (C) TPSA has anomalies concentrated at lower surface areas (15–40 A^2^). (D) Number of rings (NumRings) shows anomalies confined mostly to 0-, 1-, or 2-ring systems.

Additionally, we ran 10000 Monte Carlos simulations using the Python SciPy libraries on 211 (5 positive labels +206 negative labels) test instances to establish a probability baseline to assess whether AGENTi’s performance is due to random chance or a learned inference from the training data. At a resulting baseline of 2.37% (expected probability of correctly identifying at least three out of five true positives by chance in a sample size of 211), AGENTi offers a ∼25-fold accuracy enrichment. Using the Hypergeometry Test through SciPy, we calculated a *p*-value of 0.0044, which indicates that our performance advantage (i.e., accuracy enrichment) is statistically significant. Simply put, the probability of AGENTi correctly capturing three out of five controls by pure chance is 0.44%, and therefore, there is a 99.6% confidence that our model’s ability to achieve the 60% detection outcome for the anomaly controls is due to data pattern learning.

It is worth mentioning that while the current AGENTi implementation is applicable only for protonated species, the underlying ML framework of AGENTi is transferable to potentially other ionization adducts. For example, extending the workflow to deprotonated adducts ([M–H]) would require retraining the autoencoder and regression model on dual APCI-ESI source data using the same descriptor types. The modular architectural design of AGENTi ensures that the feature space will automatically adapt to learn the nominal descriptor parameters of deprotonated species.

## Conclusion

In summary, we have demonstrated in this article the feasibility of a prospective and high-throughput approach to assess IM-MS-relevant ionization quality for the two commonly practiced mass spectrometry ionization methods—ESI and APCI. Although the training data are very limited for building our ML models relative to most AI applications, we still managed to capture three out of five proxy anomaly controls. Although within the scope of this work a 60% positive control detection is not optimal, this still translates to a 60% reduced risk of incorrect ion assignment, given that the necessary exploratory analysis follow-up is taken. With that said, the alternative is a 0% detection rate and an absence of preventive quality control since there is a lack of an established method in action for probing ionization fidelity a priori.

However, we must highlight two explicit limitations of the current model to advance this framework beyond its pilot stage. First, the model extracts its 18 descriptors exclusively from the unprotonated, native SMILES. Consequently, the autoencoder will reconstruct the input with minimal error if an anomalous compound possesses nominal configurations in its native neutral state. This may explain the current 40% FNR. Second, ΔCCS discrepancies in highly flexible molecules can arise from differences in internal energy distributions between ESI and APCI desolvation environments rather than a protomeric shift. Higher temperature can activate high-energy barrier conformation transitions, yielding structural variance independent of charge localization. AGENTi implements a conservative 2% nominal descriptor boundary to deliberately filter out minor conformational flexibility and mitigate FPR arising from thermal fluctuations. Lastly, the current implementation is strictly configured for protonated systems. Extending the framework to deprotonated adducts or metal adducts will require separate training loops to learn the nominal descriptor parameters of those adducts.

Based on our current performance interpretations, we believe AGENTi’s robustness can be significantly improved once more dual or companion ESI-APCI data for new chemical systems become available. It is worth emphasizing that AGENTi is not only an innovative approach, but it also establishes a supportive computational screening workflow that can be used to help experimentalists anticipate structural discrepancies and maintain proactive quality control before laboratory execution.

Finally, the underlying probing scheme behind AGENTi can be applied to other detection science applications, given that relevant training data are provided. Therefore, to support the broader use of our approach, we have available, upon request, a trainable version of AGENTi, where a user can input data from multiple parallel experimental methods whereby potentially anomalous targets can be prospectively screened.

## Supplementary Material



## Data Availability

AGENTi data, documentation, and executable model are accessible at https://github.com/mitkeng/AGENTi

## Notes

GBT regression model hyperparameters and tabulated training set ΔCCS values (PDF)
