# Supplementary material for: AGENTi: APCI-ESI Analyte Ionization Fidelity Pre-Screening Workflow using Multimodel Inference
Source: J Chem Inf Model. 2026 Sep 24;66(19):12316–25. doi: 10.1021/acs.jcim.6c01085 (PMC13663852; doi:10.1021/acs.jcim.6c01085)
Supplement: Supplementary file 1 [file ci6c01085_si_001.pdf]

AGENTi: APCI-ESI Analyte Ionization Fidelity Pre-Screening Workflow using Multimodel Inference

Mithony Keng and Kenneth M Merz, Jr.*

Department of Chemistry, Michigan State University,

East Lansing, Michigan 48824, United States

Department of Biochemistry and Molecular Biology, Michigan State University,

East Lansing, Michigan 48824, United States

*Corresponding Author: Kenneth M. Merz

*Corresponding Author Email: merz@chemistry.msu.edu

Table S1. APCI and ESI experimental collision cross section (CCS) training dataset[1]. All systems have ΔCCS values < 2%.

[M+H]+ Ions	ESI CCS (Å^2)	APCI CCS (Å^2)	ΔCCS (%)	STDev.
(-)-Nicotine	137.2	136.5	0.51	0.49
1-(2-Chlorophenyl)-N-methyl-N-(1-methylpropyl)-3-isoquinolinecarboxamide	185.9	186.6	0.38	0.49
1-(o-Tolyl)biguanide	144.7	144.7	0.00	0.00
1-{Bis[3-(dimethylamino)propyl]amino}propan-2-ol	158.1	157.5	0.38	0.42
17beta-Trenbolone	166.4	166.2	0.12	0.14
1-Amino-2-methylanthraquinone	153.2	153.6	0.26	0.28
1-Benzylquinolinium chloride	148	147.6	0.27	0.28
1-Butyl-1-methylpyrrolidinium chloride	132.4	131.2	0.91	0.85
1-Butyl-3-methylimidazolium chloride	128.9	129	0.08	0.07
1-Cyclohexylpyrrolidin-2-one	140.3	139.2	0.79	0.78
1-Hydroxybenzotriazole	125.9	124.9	0.80	0.71
1-Methyl-3-phenyl-5-(3-(trifluoromethyl)phenyl)-4-pyridone	179.8	179.6	0.11	0.14
1-Octyl-2-pyrrolidone	158.5	158.6	0.06	0.07
1-Piperazineethanol	125.6	124.7	0.72	0.64

2-(2-Pyridyl)ethanol	121.2	120.7	0.41	0.35
2-(3-Phenylpropyl)pyridine	141.1	140.8	0.21	0.21
2-(8-Heptadecenyl)-2-imidazoline-1-ethanol	199.8	199.3	0.25	0.35
2-(N-Ethyl-m-toluidino)ethanol	140.6	140	0.43	0.42
2-(Pyridin-4-yl)ethanol	124.3	124.6	0.24	0.21
2-[{2-[2-(Dimethylamino)ethoxy]ethyl}(methyl)amino]ethanol	138.3	138.5	0.14	0.14
2-[4-(Diethylamino)-2-hydroxybenzoyl]benzoic acid	177.1	177.2	0.06	0.07
2-Amino-6-nitrobenzothiazole	143.6	144.1	0.35	0.35
2-Aminobenzimidazole	126.8	126.2	0.47	0.42
2-Aminobenzothiazole	128.6	127.8	0.62	0.57
2-Benzyl-2-(dimethylamino)-1-[4-(morpholin-4-yl)phenyl]butan-1-one	195.7	195.7	0.00	0.00
2-Ethyl-4-methyl-1H-imidazole	121.2	120.3	0.75	0.64
2-Isopropyl-6-methyl-4-pyrimidone	132.5	133.9	1.05	0.99
2-Isopropylthioxanthone	156.4	155.5	0.58	0.64
2-Mercaptobenzimidazole	127.8	128	0.16	0.14
2-Methyl-3(2H)-isothiazolone	117.9	117.1	0.68	0.57
2-Methyl-4'-(methylthio)-2-morpholinopropiophenone	166.7	166.4	0.18	0.21
2-Methylquinoline	126.2	125.2	0.80	0.71
2-n-Octyl-4-isothiazolin-3-one	158	158.3	0.19	0.21
2-Phenylbenzimidazole	143	142.8	0.14	0.14
2-Piperidineethanol	126.6	125.7	0.71	0.64
3-(3-Pyridyl)-1-propanol	128.4	128	0.31	0.28
3-(Aminomethyl)pyridine	119.6	118.5	0.92	0.78
3-(Ethyl(3-methylphenyl)amino)propanenitrile	144.3	144.7	0.28	0.28
3-(Octyloxy)propan-1-amine	156.2	156.9	0.45	0.49
3-[Ethyl(phenyl)amino]propanenitrile	139.3	138.5	0.58	0.57
3-{[3-(Dimethylamino)propyl]amino}propanenitrile	131.9	130.8	0.84	0.78
3'-Amino-4'-methoxyacetanilide	137.2	137.3	0.07	0.07
3-Hydroxy-2-(hydroxymethyl)pyridine hydrochloride	122.1	121.7	0.33	0.28
3-Hydroxy-2-naphthanilide	160.7	160.9	0.12	0.14
3-Hydroxy-2-naphthoic o-anisidide	167.5	167.3	0.12	0.14
3-Hydroxy-4-butyrophenetidide	155.6	155.5	0.06	0.07
3-Methyl-1-phenyl-1H-pyrazol-5-amine	136.5	136.3	0.15	0.14
3'-Methyl-4-(dimethylamino)azobenzene	161.7	161.8	0.06	0.07
3-Methyl-N-phenylaniline	143.6	143.2	0.28	0.28
3-Pyridinamine	115.2	115	0.17	0.14
4-(2-Hydroxyethyl)morpholine	125	124.7	0.24	0.21
4-(2-Phenylpropan-2-yl)-N-[4-(2-phenylpropan-2-yl)phenyl]aniline	220.3	220.2	0.05	0.07
4-(3-Phenylpropyl)pyridine	146.1	145.9	0.14	0.14
4-(Diethylamino)benzaldehyde	140.3	139.4	0.64	0.64
4-(Diethylamino)salicylaldehyde	143.7	142.6	0.77	0.78
4-(Diglycidylamino)phenyl glycidyl ether	167	167.5	0.30	0.35

4-(Dimethylamino)benzoic acid 2-ethylhexyl ester	185.2	185.9	0.38	0.49
4-(Methylnitrosamino)-1-(3-pyridyl)-1-butanol	146.4	146.4	0.00	0.00
4-Aminobenzamide	126.5	126.4	0.08	0.07
4-Dimethylaminoantipyrine	152.5	152.1	0.26	0.28
4-Dimethylaminobenzaldehyde	129	129.3	0.23	0.21
4-Dodecylmorpholine	176.3	176.3	0.00	0.00
4-Hexylaniline	162.8	162.8	0.00	0.00
4-Hexyloxyaniline	167.4	168	0.36	0.42
4-Hydroxy-3-methoxypyridine	121.8	120.7	0.91	0.78
4-Hydroxytamoxifen	200.9	201	0.05	0.07
4-Methoxy-2-methyl-N-phenylaniline	150.5	150.5	0.00	0.00
4-Methylpyridin-2-amine	119.5	118.1	1.18	0.99
4-Morpholinepropanamine	128.2	128.3	0.08	0.07
4-Morpholinepropanesulfonic acid	142.4	142.7	0.21	0.21
4-Nitrosodiphenylamine	144.2	144.3	0.07	0.07
4-Pyridinol	116.2	116	0.17	0.14
4-tert-Butylaniline	147.6	147.3	0.20	0.21
5-Methoxypsoralen	140.3	139.3	0.72	0.71
5-Methyl-1H-benzotriazole	124.8	125.1	0.24	0.21
6-(1-Methylethyl)quinoline	138.5	138	0.36	0.35
6-aminopyridine-3-carboxamide	131.3	130.2	0.84	0.78
6-Methoxy-2-benzothiazolamine	136.6	136.6	0.00	0.00
6-Methyl-3-hydroxypyridine	119.4	118.1	1.09	0.92
6-Methylpyridin-2-amine	119.5	118.6	0.76	0.64
6-Methylquinoline	125.8	125.7	0.08	0.07
6-Nitrobenzimidazole	136.5	136.5	0.00	0.00
6-Thioguanine	133.4	132.4	0.75	0.71
7-(Dimethylamino)-4-methylcoumarin	141.3	140.9	0.28	0.28
7-Diethylamino-4-methylcoumarin	152.6	152.6	0.00	0.00
8-Hydroxyquinoline	124.4	124.4	0.00	0.00
8-Hydroxyquinoline citrate	125.1	124.8	0.24	0.21
9-Aminoacridine hydrochloride monohydrate	138.9	138.8	0.07	0.07
Acetamiprid	154.6	155.1	0.32	0.35
Adenine	125.2	124.4	0.64	0.57
Albendazole	166	166.9	0.54	0.64
Alloxydim sodium	175.7	175.7	0.00	0.00
alpha-Naphthoflavone	160.7	161.7	0.62	0.71
Ametryn	155.5	155.2	0.19	0.21
Amiloride hydrochloride	150.2	149.5	0.47	0.49
Aminocarb	152.2	151.6	0.39	0.42
Amiodarone hydrochloride	224.2	223.8	0.18	0.28
Amitriptyline hydrochloride	167.5	166.8	0.42	0.49
Amoxapine	172.6	172.4	0.12	0.14
Amrinone	143.3	142.7	0.42	0.42
Anabasine	135.9	136.4	0.37	0.35
Ancymidol	164.2	164.3	0.06	0.07

Antioxidant 1098	263.3	263.2	0.04	0.07
Apomorphine hydrochloride	162.5	162.3	0.12	0.14
Atenolol	157.8	157.9	0.06	0.07
Atorvastatin	229.7	229.1	0.26	0.42
Atorvastatin calcium	230.3	229.8	0.22	0.35
Atrazine	149.5	149	0.34	0.35
Atropine sulfate monohydrate	172.2	171.6	0.35	0.42
Auramine hydrochloride	169.8	168.3	0.89	1.06
AVE2865	202.4	202.7	0.15	0.21
AVE3247	228.8	228.1	0.31	0.49
AVE3295	197.4	198	0.30	0.42
AVE5638	192.4	192.1	0.16	0.21
AVE6324	267.7	268	0.11	0.21
AVE9423	210.8	210.6	0.09	0.14
Azaconazole	165.9	166.6	0.42	0.49
Azacyclotridecan-2-one	148.8	148.2	0.40	0.42
Azamethiphos	161.5	160.6	0.56	0.64
Aziprotryne	148.9	149.1	0.13	0.14
Azoxystrobin	195	196.5	0.77	1.06
Basic Blue 7	240.6	241.9	0.54	0.92
Benalaxyl	171.8	171.7	0.06	0.07
Benfuracarb	197.3	197.3	0.00	0.00
Benodanil	146.3	146.6	0.20	0.21
Benzalkonium chloride C12 (BAC C12)	193.9	193	0.47	0.64
Benzalkonium chloride C18 (BAC C18)	216.6	215.1	0.69	1.06
Benzalkonium chloride C8 (BAC C8)	171.3	170.1	0.70	0.85
Benzethonium chloride	209.1	208.1	0.48	0.71
Benzimidazole	121.8	121	0.66	0.57
Benzo(f)quinoline	134.8	134.7	0.07	0.07
Besonprodil	203.4	203.6	0.10	0.14
Bezafibrate	177.8	178.5	0.39	0.49
Biochanin A	164.1	164.6	0.30	0.35
Biotin	149.3	149.9	0.40	0.42
Bis(4-(dimethylamino)phenyl)methanone	166.5	166.8	0.18	0.21
Bis[2-(2-butoxyethoxy)ethyl] adipate	205.4	203.8	0.78	1.13
Bispyribac-sodium	198.5	198.3	0.10	0.14
Bosentan hydrate	229.4	229.9	0.22	0.35
Bromocriptine mesylate	247.4	242.9	1.84	3.18
Brompheniramine hydrogen maleate	167.8	167.1	0.42	0.49
Bromuconazole	172.6	172.7	0.06	0.07
Brucine	194.1	191.3	1.45	1.98
Buflomedil hydrochloride	176.1	176	0.06	0.07
Bupirimate	175.8	175.8	0.00	0.00
Buprofezin	173.9	173.6	0.17	0.21
Buspirone	199.2	199.2	0.00	0.00
Butam	154.5	154.6	0.06	0.07

Buturon	153.7	152.2	0.98	1.06
C.I. Basic Blue 9	165.7	164.7	0.61	0.71
C.I. Basic Orange 2	151.2	150.7	0.33	0.35
C.I. Basic Red 9 monohydrochloride	174.1	173.3	0.46	0.57
C.I. Basic Violet 1	200	198.6	0.70	0.99
C.I. Disperse Black 6	170	170.4	0.24	0.28
C.I. Disperse Black 6 dihydrochloride	169.9	169.7	0.12	0.14
C.I. Disperse Blue 3	167.5	167.2	0.18	0.21
C.I. Disperse Orange 25	188.8	188.5	0.16	0.21
C.I. Disperse Orange 37	197.9	197.8	0.05	0.07
C.I. Disperse Red 60	178	177.7	0.17	0.21
C.I. Disperse Yellow 3	168.2	167.8	0.24	0.28
C.I. Solvent Red 140	211.3	211.8	0.24	0.35
C.I. Solvent Red 49	218.5	217.9	0.27	0.42
C.I. Solvent Red 72	185	186.2	0.65	0.85
C.I. Solvent Red 80	171.6	171.6	0.00	0.00
C.I. Solvent Yellow 1	146.9	146.1	0.55	0.57
C.I. Solvent Yellow 56	165.9	165.9	0.00	0.00
Caffeine	140.1	140.6	0.36	0.35
Candesartan cilexetil	242.2	241.9	0.12	0.21
Candoxatril	224.4	224.2	0.09	0.14
Carabersat	183.9	184.1	0.11	0.14
Carbamazepine	150.1	150.5	0.27	0.28
Carbendazim	139	138.7	0.22	0.21
Carbosulfan	188.3	188.3	0.00	0.00
Carboxin	153	151.4	1.05	1.13
Cariporide mesylate	173.6	175.1	0.86	1.06
Cerivastatin sodium	217.7	217.7	0.00	0.00
Cetirizine hydrochloride (1:2)	200.3	200	0.15	0.21
Cetylpyridinium bromide	194.3	193.4	0.46	0.64
Cetylpyridinium chloride	193.7	193.3	0.21	0.28
CH 223191	189.1	189.6	0.26	0.35
Chlordimeform	146.4	145.6	0.55	0.57
Chlorfenvinphos	170.1	168.7	0.83	0.99
Chlorhexidine	222.4	222.9	0.22	0.35
Chlorhexidine diacetate	224.1	222.8	0.58	0.92
Chlorhexidine dihydrochloride	222.9	222.7	0.09	0.14
Chloridazon	144	143.6	0.28	0.28
Chloroxuron	174.9	175.3	0.23	0.28
Chlorpheniramine maleate	164.6	164	0.37	0.42
Chlorpromazine hydrochloride	171.2	170.8	0.23	0.28
Chlorpyrifos oxon	162.7	162.3	0.25	0.28
Chrysin	154.8	155.4	0.39	0.42
CI-1018	200.9	201.9	0.50	0.71
Cinchophen	156.6	156.2	0.26	0.28
CITCO	200.2	200.6	0.20	0.28

CJ-013610	196.8	196.7	0.05	0.07
Cladribine	157	157.2	0.13	0.14
Clethodim	187.7	187.7	0.00	0.00
Clofencet-potassium	159	159.2	0.13	0.14
Clomiphene citrate (1:1)	207.3	206.9	0.19	0.28
Clomipramine hydrochloride	174.5	174.5	0.00	0.00
Clonidine hydrochloride	146.5	147.1	0.41	0.42
Cloquintocet-mexyl	181.9	182.1	0.11	0.14
Cloransulam-methyl	190.7	191	0.16	0.21
Colchicine	195.9	197	0.56	0.78
Coumatetralyl	165.6	165.7	0.06	0.07
CP-114271	181.4	181.1	0.17	0.21
CP-122721	183.3	182.7	0.33	0.42
CP-401387	205.4	206	0.29	0.42
CP-457920	183.9	184.3	0.22	0.28
CP-608039	216.2	216.1	0.05	0.07
CP-642931	190.4	191.2	0.42	0.57
CP-728663	208.2	208.4	0.10	0.14
CP-863187	177	177.3	0.17	0.21
Cresyl diphenyl phosphate	178	178.3	0.17	0.21
Crotamiton	145.7	145.3	0.27	0.28
Crufomate	165	165.2	0.12	0.14
Cyanazine	155.4	155.6	0.13	0.14
Cybutryne	162.4	162.8	0.25	0.28
Cyclocytidine	143.5	143.1	0.28	0.28
Cyclopamine	219.1	218.7	0.18	0.28
Cyclophosphamide	147.7	147.8	0.07	0.07
Cyclophosphamide monohydrate	147.6	148	0.27	0.28
Cyproconazole	170.2	170.6	0.23	0.28
Cyprodinil	153.4	153.1	0.20	0.21
Cyromazine	135.9	135.6	0.22	0.21
Cytosine	118.8	117.3	1.27	1.06
D&C Violet 2	176.7	176.9	0.11	0.14
Daidzein	155.8	155.6	0.13	0.14
Danazol	187.8	188.3	0.27	0.35
Dapsone	155	155.5	0.32	0.35
Darbufelone mesylate	188	188.3	0.16	0.21
DEET	143.6	143.5	0.07	0.07
Deflazacort	204.8	205.2	0.20	0.28
Dehydrothio-4-toluidine	153.9	153.9	0.00	0.00
Deisopropylatrazine	133.7	133.5	0.15	0.14
Demeton-S-methyl sulfone	145	144.5	0.35	0.35
Desethylatrazine	139.4	139.3	0.07	0.07
Dextromethorphan hydrobromide	166.7	166.1	0.36	0.42
Diazinon	173.7	173.5	0.12	0.14
Dibenzylamine	149.6	150.8	0.80	0.85

Diclosulam	183.5	184.6	0.60	0.78
Dicyclohexylamine	146.9	147.6	0.48	0.49
Didecyldimethylammonium chloride	209.1	208.2	0.43	0.64
Diethylmethylbenzenediamine	141.3	140.6	0.50	0.49
Difenoconazole	201.8	205	1.57	2.26
Difenzoquat metilsulfate	160.8	160	0.50	0.57
Difpas-pyrazole	187.4	187.8	0.21	0.28
Dimethametryn	165	165.4	0.24	0.28
Dimethenamid	155.9	155.2	0.45	0.49
Dimethomorph	199.2	200.7	0.75	1.06
Dimethylarsinic acid	109	108.6	0.37	0.28
Dimetilan	153.1	152.5	0.39	0.42
Diniconazole	177.1	177.9	0.45	0.57
Diphenamid	154.7	154.1	0.39	0.42
Di-p-methylbenzylidenesorbitol	193.1	191.5	0.83	1.13
Diquat dibromide monohydrate	134.4	133.6	0.60	0.57
Disperse Red 82	213.3	213.1	0.09	0.14
Diuron	150.9	151.3	0.26	0.28
Dodecyltrimethylammonium chloride	175.9	175.2	0.40	0.49
Doxorubicin hydrochloride	230	230.5	0.22	0.35
Dulcin	144.1	144.5	0.28	0.28
Econazole nitrate	185.7	185.8	0.05	0.07
Edifenphos	162.2	162	0.12	0.14
Elzasonan	203.8	204	0.10	0.14
Enadoline	191.7	191.8	0.05	0.07
Epoxiconazole	176.6	176.9	0.17	0.21
Erythromycin	257.1	258.5	0.54	0.99
Etaconazole	174.1	175.4	0.74	0.92
Ethirimol	152	152.1	0.07	0.07
Ethoprop	151.5	151.3	0.13	0.14
Ethoxyquin	153.2	153.1	0.07	0.07
Ethyl diethanolamine	125.8	125.6	0.16	0.14
Etoxazole	186.5	186.3	0.11	0.14
Fabesetron hydrochloride	174.8	175.1	0.17	0.21
Famotidine	168.9	168.7	0.12	0.14
Farglitazar	217.1	217.4	0.14	0.21
FD&C Red 3	211.4	211.7	0.14	0.21
Fenamidone	172.7	172.6	0.06	0.07
Fenamiphos	171.3	172.5	0.70	0.85
Fenamiphos sulfone	180.3	182.4	1.16	1.48
Fenarimol	176.7	177.4	0.40	0.49
Fenbendazole	174.6	175	0.23	0.28
Fenhexamid	166.4	166.7	0.18	0.21
Fenoxaprop-ethyl	189.3	190.2	0.47	0.64
Fenoxaprop-P-ethyl	189.5	189.9	0.21	0.28
Fenuron	134.4	134	0.30	0.28

Finasteride	203.3	204.7	0.69	0.99
Fipexide hydrochloride	190.3	190.4	0.05	0.07
Flavone	146.5	146.6	0.07	0.07
Florasulam	172.1	172.3	0.12	0.14
Fluazifop-butyl	195.1	195.5	0.20	0.28
Fluconazole	167.2	167.5	0.18	0.21
Flumetsulam	167.7	167.4	0.18	0.21
Fluometuron	150.1	150.5	0.27	0.28
Fluorescein	174.2	174.2	0.00	0.00
Fluorescein sodium	173.8	174.3	0.29	0.35
Fluoxastrobin	204.3	204.8	0.24	0.35
Flusilazole	174.9	176.6	0.97	1.20
Fluthiacet-methyl	188.2	188.3	0.05	0.07
Flutolanil	172.7	173.1	0.23	0.28
Forchlorfenuron	156.9	156.5	0.26	0.28
Formononetin	160.3	159.8	0.31	0.35
FR130739	196.8	197	0.10	0.14
FR150011	211.5	211.8	0.14	0.21
Fuberidazole	136.8	136.1	0.51	0.49
Fulvestrant	242.9	243.5	0.25	0.42
Furalaxyl	161.4	162.1	0.43	0.49
Furathiocarb	190.5	191.3	0.42	0.57
Genistein	160.3	160.3	0.00	0.00
Gentian Violet	205.8	205	0.39	0.57
Glipizide	200.4	201.4	0.50	0.71
Griseofulvin	181.6	182	0.22	0.28
GW473178E methyl benzene sulphonic acid	186.4	186.4	0.00	0.00
Haloperidol	195.1	195.1	0.00	0.00
HC Blue 2	171.1	171.3	0.12	0.14
HC Red 3	142.2	141.6	0.42	0.42
Hexaconazole	173.5	174.6	0.63	0.78
Hexadecyltrimethylammonium bromide	195.1	193.1	1.03	1.41
Hexamethylphosphoramide	135.3	134.4	0.67	0.64
Hexazinone	162.8	163	0.12	0.14
HMR1171 trifluoroacetate (1:1)	249.1	249.1	0.00	0.00
HMR1426	167.9	167.8	0.06	0.07
Hydramethylnon	240.6	239.4	0.50	0.85
Hydroxyzine hydrochloride	197.2	197.7	0.25	0.35
Ifosfamide	148.8	148.1	0.47	0.49
Ilepatril	194.9	195.4	0.26	0.35
Imazalil	167.9	167.9	0.00	0.00
Imazamethabenz-methyl	168.2	168.1	0.06	0.07
Imazamox	172.4	172.2	0.12	0.14
Imazapic	162.4	162.2	0.12	0.14
Imazapyr	156.7	156.5	0.13	0.14
Imazaquin	171.7	171.7	0.00	0.00

Imazethapyr	168	168.1	0.06	0.07
Imipramine hydrochloride	166	165.8	0.12	0.14
Indinavir sulfate	262.3	262.9	0.23	0.42
Indoxacarb	205.7	206.6	0.44	0.64
Ingliforib	216	215.9	0.05	0.07
Iodosulfuron methyl ester sodium salt	194.2	194.5	0.15	0.21
Ipconazole	173.8	173.8	0.00	0.00
Isopropyl triethanolamine titanate	118.9	119.2	0.25	0.21
Isoproturon	152.1	152.9	0.52	0.57
Isoxaben	185.4	186.1	0.38	0.49
Kaempferol	162.8	163.2	0.25	0.28
Ketamine hydrochloride	149.4	149.7	0.20	0.21
Ketoconazole	216.8	217.3	0.23	0.35
Khellin	151.1	151.6	0.33	0.35
Kinetin	144.4	144.3	0.07	0.07
Labetalol hydrochloride	175.2	175.7	0.28	0.35
Laurocapram	184.7	185.5	0.43	0.57
Lauryldiethanolamine	184.1	183.7	0.22	0.28
Lepidine	125.4	126.3	0.72	0.64
Leucomalachite green	198.9	199.9	0.50	0.71
Levamisole hydrochloride	144.8	144.2	0.42	0.42
Loratadine	187.4	187.9	0.27	0.35
Losartan potassium	201.2	201.6	0.20	0.28
Malachite green	188.9	188.5	0.21	0.28
Maprotiline hydrochloride	164.3	164.1	0.12	0.14
Megestrol acetate	197.2	198.2	0.51	0.71
Melatonin	153.3	153.7	0.26	0.28
Melengestrol acetate	200.3	201.5	0.60	0.85
Meloxicam	178.5	179.2	0.39	0.49
Mepanipyrim	153.1	152.6	0.33	0.35
Mepronil	169.4	169.9	0.29	0.35
Metalaxyl	160.8	160.5	0.19	0.21
Metalaxyl-M	160	160.5	0.31	0.35
Metamitron	145.2	144.9	0.21	0.21
Metconazole	169.1	169.3	0.12	0.14
Metepa	143.2	142.5	0.49	0.49
Metformin	125.7	125.1	0.48	0.42
Methabenzthiazuron	144.7	144.2	0.35	0.35
Methadone hydrochloride	176.7	176.3	0.23	0.28
Methapyrilene hydrochloride	157.6	157.6	0.00	0.00
Methfuroxam	153.3	152.5	0.52	0.57
Methimazole	118.1	116.9	1.02	0.85
Methiuron	143.3	143.1	0.14	0.14
Methyl red	163	163.1	0.06	0.07
Methyltrioctylammonium chloride	221.8	222.2	0.18	0.28
Metolachlor	159.8	158.9	0.56	0.64

Metoprolol	172.1	171.7	0.23	0.28
Metribuzin	148.4	146.7	1.15	1.20
Metribuzin-DA	145.2	144.2	0.69	0.71
Metronidazole	134.3	133.8	0.37	0.35
Milrinone	156.3	157.1	0.51	0.57
Minocycline hydrochloride	210.1	209.5	0.29	0.42
Mizolastine	203.7	204.1	0.20	0.28
MK-547	230.9	231	0.04	0.07
MK-578	243.4	243.1	0.12	0.21
MK-968	187.7	187.8	0.05	0.07
Monocrotaline	175.3	175.4	0.06	0.07
Monuron	143	142.7	0.21	0.21
Mosapride citrate	194.6	195.8	0.61	0.85
Myristyltrimethylammonium chloride	185.7	185.2	0.27	0.35
N-(3-Aminophenyl)propanamide	136.5	135.8	0.51	0.49
N-(3-Dimethylaminopropyl)methacrylamide	137.1	135.8	0.95	0.92
N-(4-Nitrophenyl)-N'-(3-pyridinylmethyl)urea	174.3	174.7	0.23	0.28
N-[3-(Dimethylamino)propyl]dodecanamide	185.2	185.5	0.16	0.21
N2-(4-Nitrophenyl)-L-glutamine hydrochloride(1:1)	170.8	171.8	0.58	0.71
Nalidixic acid	147.8	147.5	0.20	0.21
Napropamide	164.2	163.8	0.24	0.28
N-Benzyl-1H-purin-6-amine	148.3	148.7	0.27	0.28
N-Benzyl-9-(tetrahydro-2H-pyran-2-yl)adenine	176.5	177.9	0.79	0.99
N-Butyldiethanolamine	136.7	136.3	0.29	0.28
Nelivaptan	230.6	231.8	0.52	0.85
N-Ethyl-2-(isopropyl)-5-methylcyclohexanecarboxamide	152.7	153.5	0.52	0.57
N-Ethylphthalimide	135.4	133.7	1.26	1.20
Nicardipine hydrochloride	212.6	213	0.19	0.28
Nicotine sulfate (2:1)	137.7	137	0.51	0.49
Nicotinyl alcohol	119.8	119.2	0.50	0.42
Nizatidine	171	171.3	0.18	0.21
N-methyl-alpha-phenylbenzeneacetamide	152.9	153.4	0.33	0.35
N-Methyl-N-nitrosoaniline	125.1	124.5	0.48	0.42
Nomifensine	156.2	155.8	0.26	0.28
Norflurazon	162.4	162.7	0.18	0.21
N-Phenyl diethanolamine	137.9	137.7	0.15	0.14
N-Phenyl-2-naphthylamine	151.3	150	0.86	0.92
Nuarimol	171.2	171.6	0.23	0.28
o-Aminoazotoluene	156	155.9	0.06	0.07
Octabenzone	192.8	192.5	0.16	0.21
Oleyl sarcosine	200.6	200.7	0.05	0.07
Oxazepam	163.7	163.5	0.12	0.14
Oxcarbazepine	154.8	153.9	0.58	0.64
Oxfendazole	178.2	179	0.45	0.57
Oxolinic acid	152.3	152.6	0.20	0.21

Oxytetracycline hydrochloride	197.8	199.9	1.06	1.48
Panthenol	145.3	145	0.21	0.21
Pantoprazole sodium	185.2	185.5	0.16	0.21
Parbenate	149.6	149.3	0.20	0.21
Parinol	183.1	183.7	0.33	0.42
PD 0343701	202.4	202.2	0.10	0.14
PD-0333941	190.6	190.8	0.10	0.14
Penconazole	166.1	166.3	0.12	0.14
Pencycuron	180.5	180.7	0.11	0.14
Penicillin VK	182.1	182	0.05	0.07
Penoxsulam	195.6	196	0.20	0.28
PHA-00568487	175.7	175.9	0.11	0.14
PharmaGSID_47263	170.3	170.8	0.29	0.35
PharmaGSID_47330	196.4	197.1	0.36	0.49
PharmaGSID_47333	192.4	192.3	0.05	0.07
PharmaGSID_47337	228.8	228.4	0.17	0.28
PharmaGSID_48166	192.1	191.3	0.42	0.57
PharmaGSID_48172	213.7	214.3	0.28	0.42
PharmaGSID_48505	180	180.2	0.11	0.14
PharmaGSID_48507	192.4	192.8	0.21	0.28
PharmaGSID_48510	200.4	201.3	0.45	0.64
PharmaGSID_48513	244.6	245.7	0.45	0.78
PharmaGSID_48519	218.9	219.5	0.27	0.42
PharmaGSID_48521	244.1	245	0.37	0.64
Phenacetin	141.5	141.4	0.07	0.07
Phenazone	139	138.9	0.07	0.07
Phenazopyridine hydrochloride	150.3	150	0.20	0.21
Phenolphthalein	173.9	173.8	0.06	0.07
Phosfolan	150.2	150.2	0.00	0.00
Phosphamidon	160.7	160.5	0.12	0.14
Physostigmine	169.8	169.6	0.12	0.14
Pimozide	211.8	211.9	0.05	0.07
Pinoxaden	196.6	196.5	0.05	0.07
Pioglitazone hydrochloride	178.9	178.8	0.06	0.07
Piperine	175.7	176	0.17	0.21
Pirimiphos-ethyl	178.3	176.3	1.13	1.41
Pirimiphos-methyl	167.9	165.5	1.44	1.70
Pitavastatin calcium	203.2	202.3	0.44	0.64
Pramipexole dihydrochloride monohydrate	152.6	152.8	0.13	0.14
Praziquantel	178.3	178.5	0.11	0.14
Procaine hydrochloride	157.1	155.9	0.77	0.85
Prochloraz	183.4	183.8	0.22	0.28
Procyazine	159	159.4	0.25	0.28
Proflavin hydrochloride	143.9	143.4	0.35	0.35
Promethazine hydrochloride	163.7	163.3	0.24	0.28
Prometon	156.5	156.1	0.26	0.28

Prometryn	161	160.9	0.06	0.07
Propachlor	143.2	143.4	0.14	0.14
Propamocarb	143.3	142.9	0.28	0.28
Propamocarb hydrochloride	143.4	142.7	0.49	0.49
Propiconazole	179.8	180.9	0.61	0.78
Propranolol hydrochloride	162.8	162.5	0.18	0.21
p-Xylenol blue	194.5	194.4	0.05	0.07
Pymetrozine	154.2	154.8	0.39	0.42
Pyraclostrobin	183.8	184.5	0.38	0.49
Pyrazophos	191.7	192.5	0.42	0.57
Pyridalyl	203.8	203	0.39	0.57
Pyridoxine	134.6	133.7	0.67	0.64
Pyrimethamine	159.4	158.8	0.38	0.42
Pyrimethanil	143.8	143.4	0.28	0.28
Pyriproxyfen	184.3	184.3	0.00	0.00
Pyrithiobac	166.7	167.1	0.24	0.28
Pyrithiobac sodium	166.6	166.7	0.06	0.07
Quaternium 17	197.2	196.1	0.56	0.78
Quetiapine fumarate	192.3	192.1	0.10	0.14
Quinalphos	162.9	162.4	0.31	0.35
Quinine hydrochloride dihydrate	178	178.8	0.45	0.57
Quinmerac	140.7	139.6	0.78	0.78
Quinoxyfen	165.5	165.8	0.18	0.21
Quizalofop-ethyl	191.4	191.5	0.05	0.07
Quizalofop-P-ethyl	191.3	192.1	0.42	0.57
Raloxifene hydrochloride	215.2	215.7	0.23	0.35
Ranitidine	169	169.2	0.12	0.14
Rhodamine 6G	218.9	218.3	0.27	0.42
Ricinine	132.4	132.2	0.15	0.14
Ro 23-7637	238	239.1	0.46	0.78
Ro 3280	250.7	250.9	0.08	0.14
Rosiglitazone maleate	172.5	172.4	0.06	0.07
Roxithromycin	276.9	277.7	0.29	0.57
SAR 102608.00	184.3	184.3	0.00	0.00
SAR 115740.00	195.7	195.9	0.10	0.14
SAR 377142.00	235.6	234.1	0.64	1.06
Sarafloxacin	188.3	188.2	0.05	0.07
SB236057A	229.1	229.6	0.22	0.35
SB243213A	213.8	214.2	0.19	0.28
SB281832	197.1	197.6	0.25	0.35
SB413217A	224.8	225.5	0.31	0.49
Scopolamine hydrobromide	171.7	170.8	0.53	0.64
Scopolamine hydrochloride	171.7	172.2	0.29	0.35
Secbumeton	155.2	155.3	0.06	0.07
Sethoxydim	181	181.6	0.33	0.42
Siduron	158	157.9	0.06	0.07

Simazine	144	143.3	0.49	0.49
Simetryn	149.5	149.6	0.07	0.07
S-Metolachlor	160.1	159.5	0.38	0.42
Sodium nicotinate	124.5	123.5	0.81	0.71
Sodium warfarin	167	167.1	0.06	0.07
Spirotetramat	189.3	190.2	0.47	0.64
SR125047	185.6	185.2	0.22	0.28
SR144190	241.6	241.2	0.17	0.28
SR146131 trifluoroacetate (1:1)	258.2	258.5	0.12	0.21
SR271425	197	196.9	0.05	0.07
SR58611	192.2	192.3	0.05	0.07
SSR 240612	267	266.1	0.34	0.64
SSR 241586 HCl	245.5	244.9	0.24	0.42
SSR146977	252.5	252.9	0.16	0.28
SSR161421	206.7	207	0.15	0.21
SSR162369	176.9	176.9	0.00	0.00
SSR504734	190.4	190.3	0.05	0.07
SSR69071	226.9	228	0.48	0.78
Strychnine	175.2	172	1.84	2.26
Strychnine hemisulphate salt	175.8	172.6	1.84	2.26
SU-5416	154.2	153.9	0.19	0.21
Sulfamethazine	163.4	163.6	0.12	0.14
Sulfaquinoxaline	167.8	168.4	0.36	0.42
Sulfasalazine	203.6	203.9	0.15	0.21
Sulfathiazole	153.9	153.5	0.26	0.28
Sulfathiazole sodium	153.6	153.3	0.20	0.21
Sulpiride	185.3	186	0.38	0.49
Surinabant	224.1	224.8	0.31	0.49
Suxibuzone	200.8	201.3	0.25	0.35
Tamoxifen	196.7	196.8	0.05	0.07
Tebuconazole	166.8	167.5	0.42	0.49
Tebufenpyrad	193	193.9	0.47	0.64
Tebupirimfos	177.4	177.4	0.00	0.00
Tebuthiuron	150.5	150.4	0.07	0.07
Temephos	189.5	189.4	0.05	0.07
Terbumeton	154.9	154.8	0.06	0.07
Terbutryn	159.4	159.9	0.31	0.35
Terbutylazine	153.8	153.8	0.00	0.00
Tetrabutylammonium bromide	168	167.2	0.48	0.57
Tetraconazole	176.6	177.6	0.56	0.71
Tetradecylpyridinium bromide	185.9	185.4	0.27	0.35
Tetradonium bromide	185.7	185.1	0.32	0.42
Tetrapropylammonium bromide	146.2	146	0.14	0.14
Theophylline	137.3	136.3	0.73	0.71
Thiabendazole	139.7	141.6	1.35	1.34
Thiamine thiozole	126.5	126.1	0.32	0.28

Thiazopyr	182.1	183.2	0.60	0.78
Thidiazuron	147.9	148.4	0.34	0.35
Thiophanate	186.6	187	0.21	0.28
Thiophanate-methyl	174.8	175.2	0.23	0.28
Thiotepa	132.5	132.5	0.00	0.00
Ticlopidine hydrochloride	156.8	156.4	0.26	0.28
Timolol maleate salt	176.5	176.1	0.23	0.28
Todralazine hydrochloride	151.8	152	0.13	0.14
Tolazamide	175.4	175.5	0.06	0.07
Tolnaftate	175.4	175.5	0.06	0.07
Tolyltriazole	125.3	124.5	0.64	0.57
Tralkoxydim	187	187.3	0.16	0.21
Tramadol	161.1	160.6	0.31	0.35
Trazodone hydrochloride	192	192.5	0.26	0.35
Trelanserin	202.8	200.1	1.34	1.91
Triadimefon	173.6	174.6	0.57	0.71
Triamterene	158.3	158.4	0.06	0.07
Triazophos	171.4	171.2	0.12	0.14
Tribenzylamine	169.2	169.1	0.06	0.07
Tribufos	177.2	177.5	0.17	0.21
Tributyltetradecylphosphonium chloride	224.5	224	0.22	0.35
Triethanolamine	129.6	129.3	0.23	0.21
Triethyl phosphonoacetate	143.7	143.6	0.07	0.07
Trifloxystrobin	187.2	187.4	0.11	0.14
Triflumizole	180	179.8	0.11	0.14
Triisopropanolamine	141.5	141.3	0.14	0.14
Tri-o-cresyl phosphate	182.3	182.7	0.22	0.28
Triphenylphosphine oxide	162.9	163	0.06	0.07
Tris(2-butoxyethyl) phosphate	195.7	196	0.15	0.21
Tris(4-methylphenyl) phosphate	190.1	191	0.47	0.64
Tris(methylphenyl) phosphate	189.7	189.4	0.16	0.21
Triticonazole	180.6	181.7	0.61	0.78
Trixylyl phosphate	203.1	204.2	0.54	0.78
UK-156819	176.7	176.6	0.06	0.07
UK-337312	262.3	260.7	0.61	1.13
UK-343664	236.5	237	0.21	0.35
UK-416244	195.7	196.5	0.41	0.57
Vatalanib dihydrochloride	187.6	188	0.21	0.28
Venlafaxine hydrochloride	170.7	170.8	0.06	0.07
Vincamine	183.2	182.5	0.38	0.49
Volinanserin	185	185	0.00	0.00
Warfarin	166.9	167.4	0.30	0.35
Xanthinol niacinate	171.7	171.8	0.06	0.07
Ximelagatran	208.6	208.6	0.00	0.00
YM 218	239	240.1	0.46	0.78
Zamifenacin	192.4	192.3	0.05	0.07

**Average**		0.3	0.4

Figure S1. Hyperparameters setting for the GBT regression model used in predicting $P_{\Delta CCS}$ (label) between APCI CCS and ESI CCS.

```
num_trees=300,
growing_strategy="BEST_FIRST_GLOBAL",
max_depth=learn_depth(charge_mode),
sampling_method="RANDOM",
weights=weight_option(charge_mode),
split_axis="SPARSE_OBLIQUE",
categorical_algorithm="RANDOM"
```

REFERENCE

(1) Teri, D.; Aly, N. A.; Dodds, J. N.; Zhang, J.; Thiessen, P. A.; Bolton, E. E.; Joseph, K. M.; Williams, A. J.; Schymanski, E. L.; Rusyn, I.; et al. Reference library for suspect screening of environmental toxicants using ion mobility spectrometry-mass spectrometry. *Communications Chemistry* **2025**, *8* (1), 224. DOI: 10.1038/s42004-025-01619-7.
